# Supplementary material for: The vestibulospinal nucleus is a locus of balance development
Source: bioRxiv. 2024 Mar 21:2023.12.06.570482. Originally published 2023 Dec 7. Preprint. [Version 2] doi: 10.1101/2023.12.06.570482 (PMC10723429; doi:10.1101/2023.12.06.570482)
Supplement: Supplement 1 [file NIHPP2023.12.06.570482v2-supplement-1.pdf]

bioRxiv preprint doi: https://doi.org/10.1101/2023.12.06.570482; this version posted March 21, 2024. The copyright holder for this preprint (which was not certified by peer review) is the author/funder, who has granted bioRxiv a license to display the preprint in perpetuity. It is made available under aCC-BY 4.0 International license.

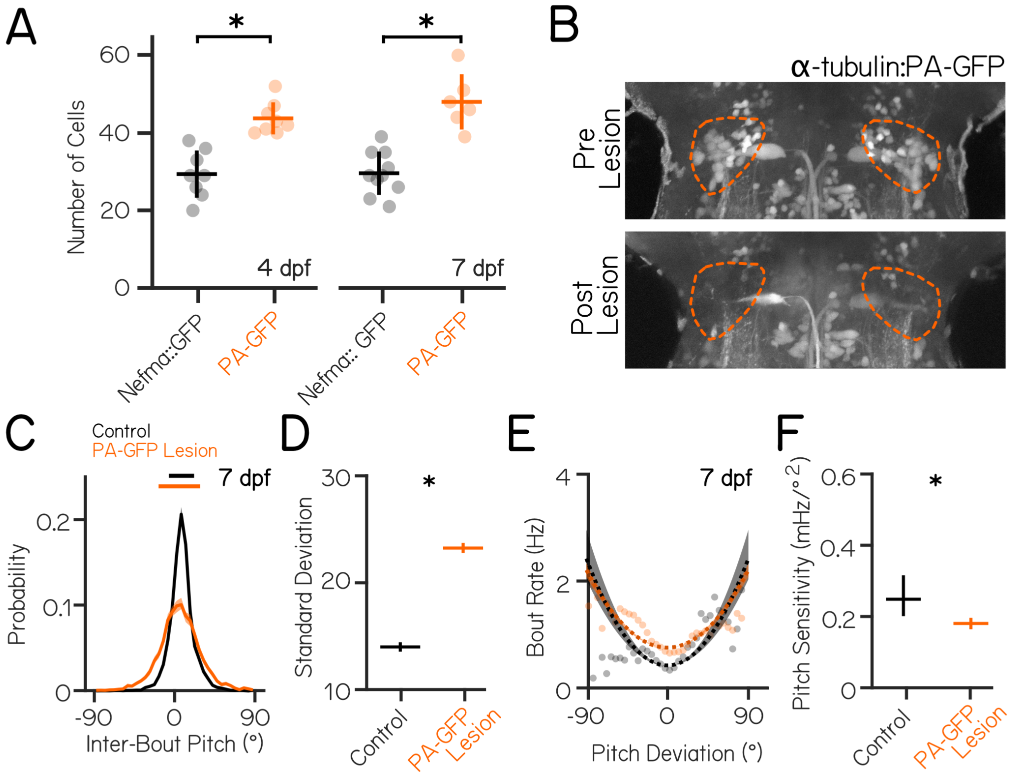

Figure S1: Lesions of a larger pool of vestibulospinal neurons at 7 dpf replicates postural disruption observed in *Tg(nefma::EGFP)* lesions. (A) *Tg(nefma::EGFP)* labels a fraction of the total vestibulospinal population labeled by optically-backfilled *Tg(α-tubulin:C3PA-GFP)* fish when measured at both 4 dpf (29±6 cells per fish nefma::EGFP vs. 44±4 cells per fish PA-GFP, p=0.0001) and 7 dpf (30±6 nefma::EGFP vs. 48±7 PA-GFP, $p=5.6\times10^{-6}$). Two-Way ANOVA revealed a significant effect of labeling strategy ($F_{1,31}=63.6$, $p=1.1\times10^{-8}$), but not of age ($F_{1,31}=1.2$, p=0.29), on number of vestibulospinal cells labeled with no significant interaction effect ($F_{1,31}=0.96$, p=0.34). Dots represent individual fish. Lines represent mean ± 1 S.D. Asterisks represent statistically significant differences in Tukey's post-hoc tests, p<0.05.
(B) Representative maximum intensity projection of spinal projecting neurons in the hindbrain of a 7 dpf *Tg(α-tubulin:C3PA-GFP)* fish following spinal photoconversions before (top) and after (bottom) two-photon mediated photoablation.
(C) Probability distributions of inter-bout pitch angle for sibling controls (black, N=17 fish) and vestibulospinal lesioned fish (orange, N=17 fish) show no change in average posture but greater variability (solid horizontal lines median ± 1 S.D.) Solid distribution lines represent probability distribution across all bouts in that condition (n=6,510/7,286 bouts Control/Lesion). Shaded error bars represents 95% confidence intervals from bootstrapped estimates of probability distributions.
(D) Standard deviation of pitch is higher in vestibulospinal lesioned fish (orange) compared to sibling controls (black). Lines are population standard deviation, error bars represent bootstrapped estimate of 95% confidence intervals. Asterisks represent statistically significant differences, p<0.05.
(E) Bout rate as a function of deviation from preferred posture for lesions (orange) and control siblings (black). Solid lines represent raw data, dashed lines represent parabolic fits to raw data, shaded error bars represents 95% confidence intervals from bootstrapped estimates of parabolic fits.
(F) Pitch sensitivity (parabolic fit) is decreased in vestibulospinal lesioned fish compared to sibling controls. Error bars represent bootstrapped estimate of 95% confidence intervals.

bioRxiv preprint doi: https://doi.org/10.1101/2023.12.06.570482; this version posted March 21, 2024. The copyright holder for this preprint (which was not certified by peer review) is the author/funder, who has granted bioRxiv a license to display the preprint in perpetuity. It is made available under aCC-BY 4.0 International license.

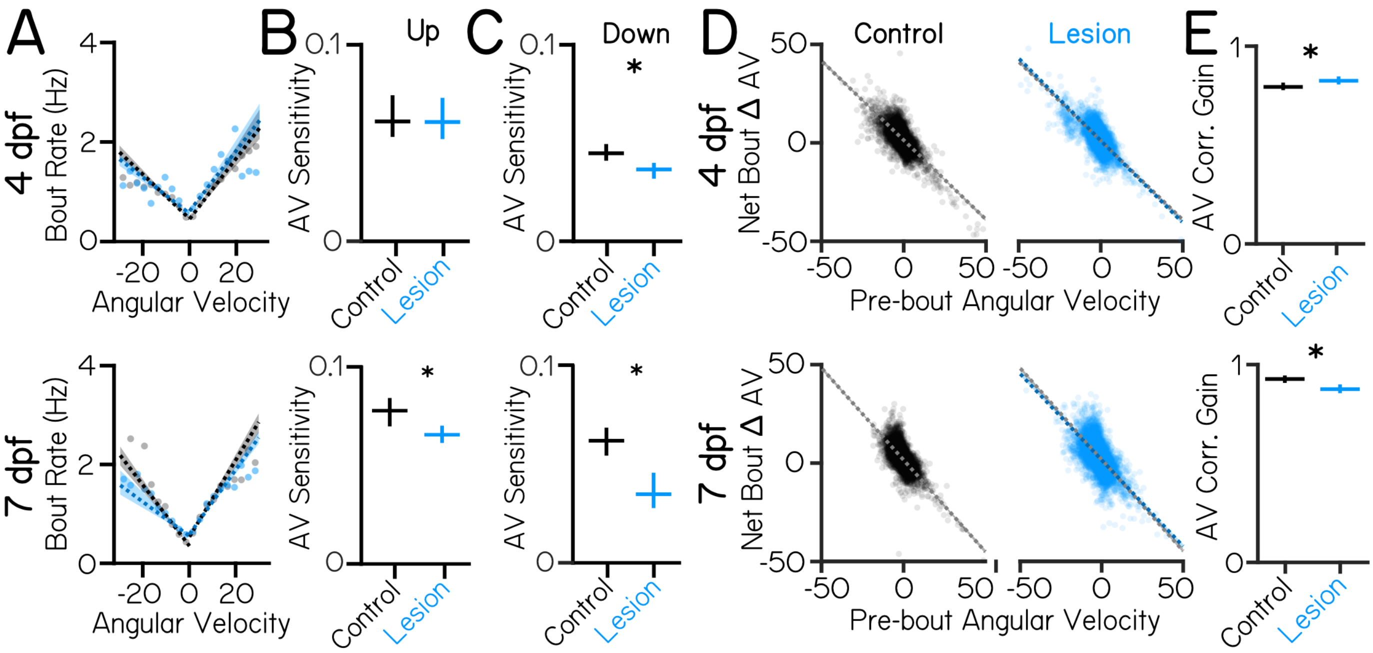

Figure S2: Vestibulospinal lesioned fish have disrupted angular velocity sensitivity and angular velocity correction. (A) Instantaneous bout rate as a function of angular velocity during inter-bout periods in vestibulospinal lesioned (blue) and control siblings (black) at 4 and 7 dpf. Dashed lines represent linear fits to raw data constrained to bouts either below or above the median angular velocity (4 dpf n=6,774/7,320 down bouts, n=6,774/7,230 up bouts Control/Lesion; 7 dpf n=6,691/9,209 down bouts, n=6,691/9,210 up bouts Control/Lesion). Shaded error bars represents 95% confidence intervals from bootstrapped estimates of linear fits. Dots represent binned means of raw data in 3°/s wide bins.

(B) Angular velocity (AV) sensitivity (magnitude of linear slope fit) for lesioned and control fish for nose-up and

(C) nose-down angular velocities.

(D) Net change in angular velocity from the beginning to end of a swim bout as a function of pre-bout angular velocity. Dashed lines represent linear fits to raw data for lesioned (blue, right) or control (black, left) fish. Shaded error bars represents 95% confidence intervals from bootstrapped estimates of linear fits. Dots are individual bouts (4 dpf n=10,225/10,725 bouts Control/Lesion; 7 dpf n=9,625/12,368 bouts Control/Lesion).

(E) Angular velocity correction gain (magnitude of linear slope fit) for lesioned and control fish at 4 and 7 dpf. Error bars in panels B, C, and E represent bootstrapped estimate of 95% confidence intervals. Asterisks represent statistically significant effects, $p<0.05$.

bioRxiv preprint doi: https://doi.org/10.1101/2023.12.06.570482; this version posted March 21, 2024. The copyright holder for this preprint (which was not certified by peer review) is the author/funder, who has granted bioRxiv a license to display the preprint in perpetuity. It is made available under aCC-BY 4.0 International license.

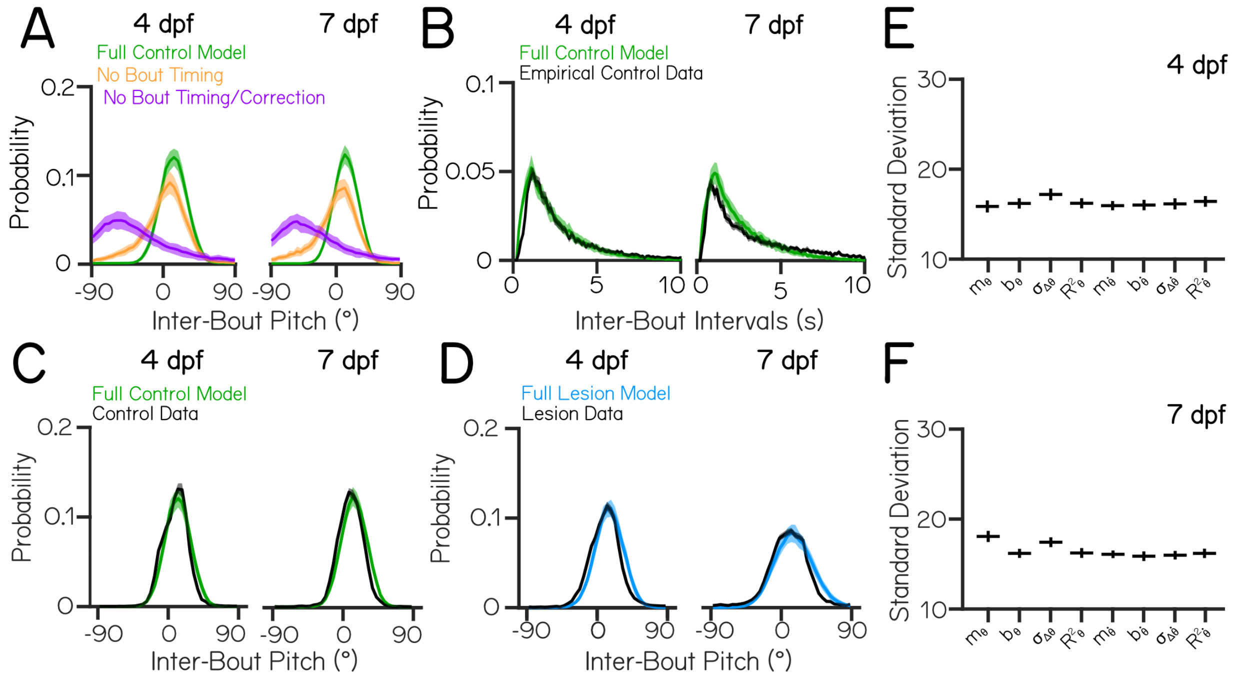

Figure S3: A model of zebrafish swimming during development generates bouts with expected pitch and event-frequency distributions. (A) Probability distributions of simulated inter-bout pitch angles in full control model (green), bout timing null model (purple), and full null model (orange) are comparable for modeling data from 4 and 7 dpf, and are comparable to previous models of larval swimming[29].
(B) Probability distributions of simulated inter-bout intervals for empirical control data (black) and simulated control fish (green) using parameters derived from 4 and 7 dpf sibling control swimming datasets.
(C) Probability distributions of observed inter-bout pitch angles at 4 and 7 dpf for empirical control data (black) and simulated control fish (green) and
(D) empirical lesion data (black) and simulated lesion fish (blue). For simulated data in panels A-D, lines are probability distribution across all 30 simulated model runs, shaded error bars ± 1 S.D. across simulated runs. For empirical data in panels A-D, lines are probability distribution across all observations per condition, shaded error bars represent bootstrapped estimates of 95% confidence intervals.
(E) Standard deviation of simulated inter-bout pitches in hybrid models with all control parameters except for a single parameter derived from lesion data at 4 dpf or
(F) 7 dpf. Parameters shown here calculate pitch bout correction (m_Θ,b_Θ, $\sigma_{\Delta\Theta}$, $R^2{}_\Theta$) and angular velocity bout correction ($m_{\dot\Theta}$,$b_{\dot\Theta}$, $\sigma_{\Delta\dot\Theta}$, $R^2{}_{\dot\Theta}$) (See Methods, "Behavioral Modeling" for additional explanation of parameters).
