# Supplementary material for: The vestibulospinal nucleus is a locus of balance development
Source: bioRxiv. 2023 Dec 7:2023.12.06.570482. Preprint. [Version 1] doi: 10.1101/2023.12.06.570482 (PMC10723429; doi:10.1101/2023.12.06.570482)
Supplement: Supplementary file 1 [file NIHPP2023.12.06.570482V1-supplement-1.pdf]

bioRxiv preprint doi: https://doi.org/10.1101/2023.12.06.570482; this version posted December 7, 2023. The copyright holder for this preprint (which was not certified by peer review) is the author/funder, who has granted bioRxiv a license to display the preprint in perpetuity. It is made available under aCC-BY 4.0 International license.

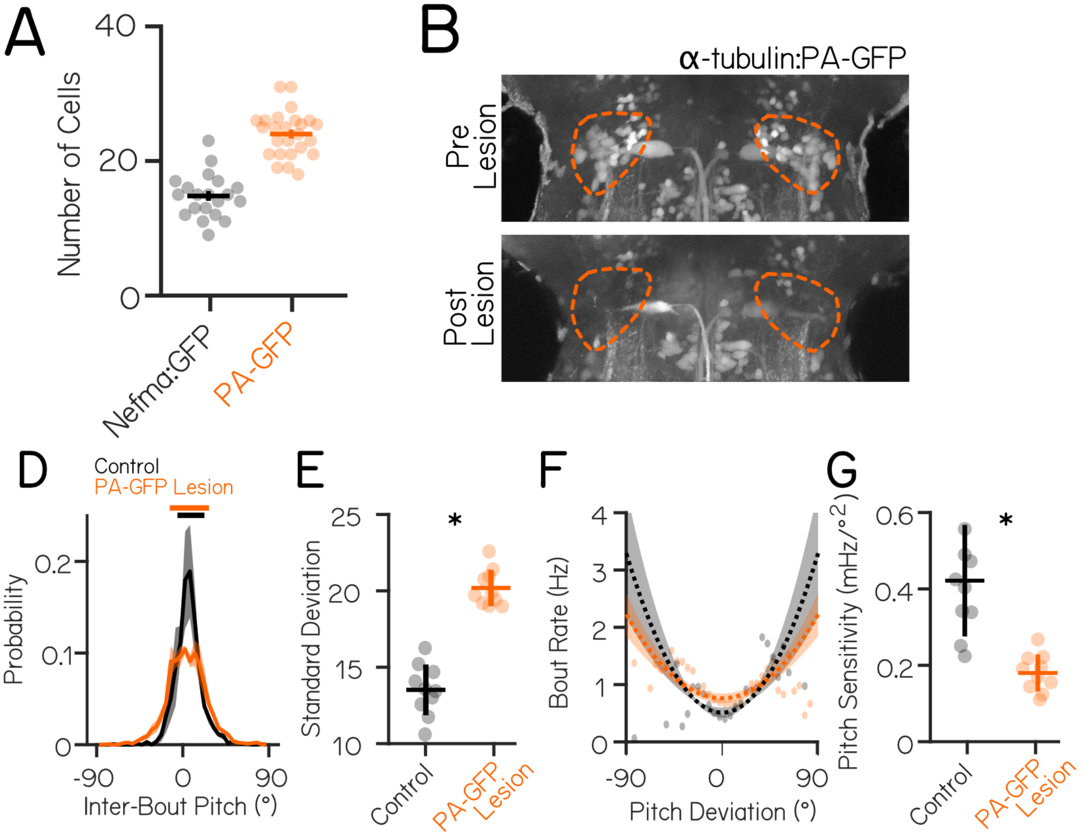

Figure S1: Lesions of a larger pool of vestibulospinal neurons at 7 dpf replicates postural disruption observed in *Tg(nefma::EGFP)* lesions. (A) *Tg(nefma::EGFP)* labels fewer (14.8 ± 0.7, n=20 hemispheres, 10 fish) neurons than optically-backfilled *Tg(α-tubulin:C3PA-GFP)* fish (24 ± 0.6, n=25 hemispheres, 13 fish, unpaired t-test $p=8.3\times10^{-12}$).
(B) Dorsoventral distribution of vestibulospinal cells in both lines. *Tg(nefma::EGFP)* labels fewer cells in the dorsal vestibulospinal nucleus compared to photofills in *Tg(α-tubulin:C3PA-GFP)*.
(C) Representative maximum intensity projection of spinal projecting neurons in the hindbrain of a *Tg(α-tubulin:C3PA-GFP)* fish following optical backfill before (top) and after (bottom) two-photon mediated photoablation.
(D) Average probability distributions (± S.D) of inter-bout pitch angle for sibling controls (black, N=17 fish) and vestibulospinal lesioned fish (orange, N=17 fish) show no change in average posture (dashed vertical lines) but greater variability (solid horizontal lines ± 1 S.D.).
(E) Standard deviation of pitch is higher in vestibulospinal lesioned fish (20.2±1.2°) compared to sibling controls (13.5 ±1.7°, unpaired t-test $p=5.5\times10^{-9}$).
(F) Bout rate as a function of deviation from preferred posture for lesions (orange) and control siblings (black). Solid lines represent raw data, dashed lines represent parabolic fits to raw data ± S.D. of the fit estimates.
(G) Pitch sensitivity (parabolic fit) is decreased in vestibulospinal lesioned fish (0.18 ± 0.05, unpaired t-test $p=9.9\times10^{-5}$) compared to sibling controls (0.42 ± 0.15).

bioRxiv preprint doi: https://doi.org/10.1101/2023.12.06.570482; this version posted December 7, 2023. The copyright holder for this preprint (which was not certified by peer review) is the author/funder, who has granted bioRxiv a license to display the preprint in perpetuity. It is made available under aCC-BY 4.0 International license.

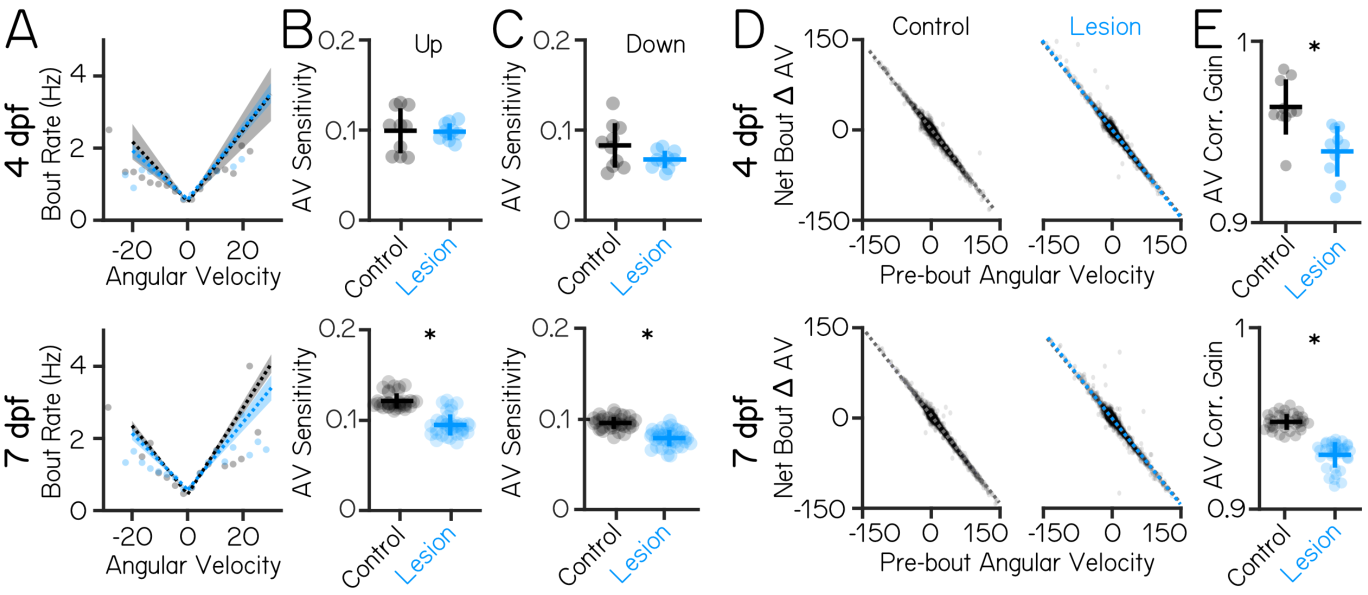

Figure S2: Vestibulospinal lesioned fish have disrupted angular velocity sensitivity and angular velocity correction. (A) Instantaneous bout rate as a function of angular velocity during inter-bout periods in vestibulospinal lesioned (blue) and control siblings (black) at 4 and 7 dpf. Dashed lines represent linear fits to raw data constrained to up and down angular velocities ±. 1 S.D. of fit estimates. Dots represent binned means of raw data in 3°/s wide bins.
(B) Angular velocity (AV) sensitivity (magnitude of linear slope fit) for lesioned and control fish for nose-up and
(C) nose-down angular velocities.
(D) Net change in angular velocity from the beginning to end of a swim bout as a function of pre-bout angular velocity. Dashed lines represent linear fits to raw data for lesioned (blue, right) or control (black, left) fish. Dots are raw data across all fish.
(E) Angular velocity correction gain (magnitude of linear slope fit) for lesioned and control fish at 4 and 7 dpf. Dots in panels B, C, and E are jackknifed estimates, lines are mean ± 1 S.D. of jackknifed estimates. Asterisks represent statistically significant effects, $p<0.05$.

bioRxiv preprint doi: https://doi.org/10.1101/2023.12.06.570482; this version posted December 7, 2023. The copyright holder for this preprint (which was not certified by peer review) is the author/funder, who has granted bioRxiv a license to display the preprint in perpetuity. It is made available under aCC-BY 4.0 International license.

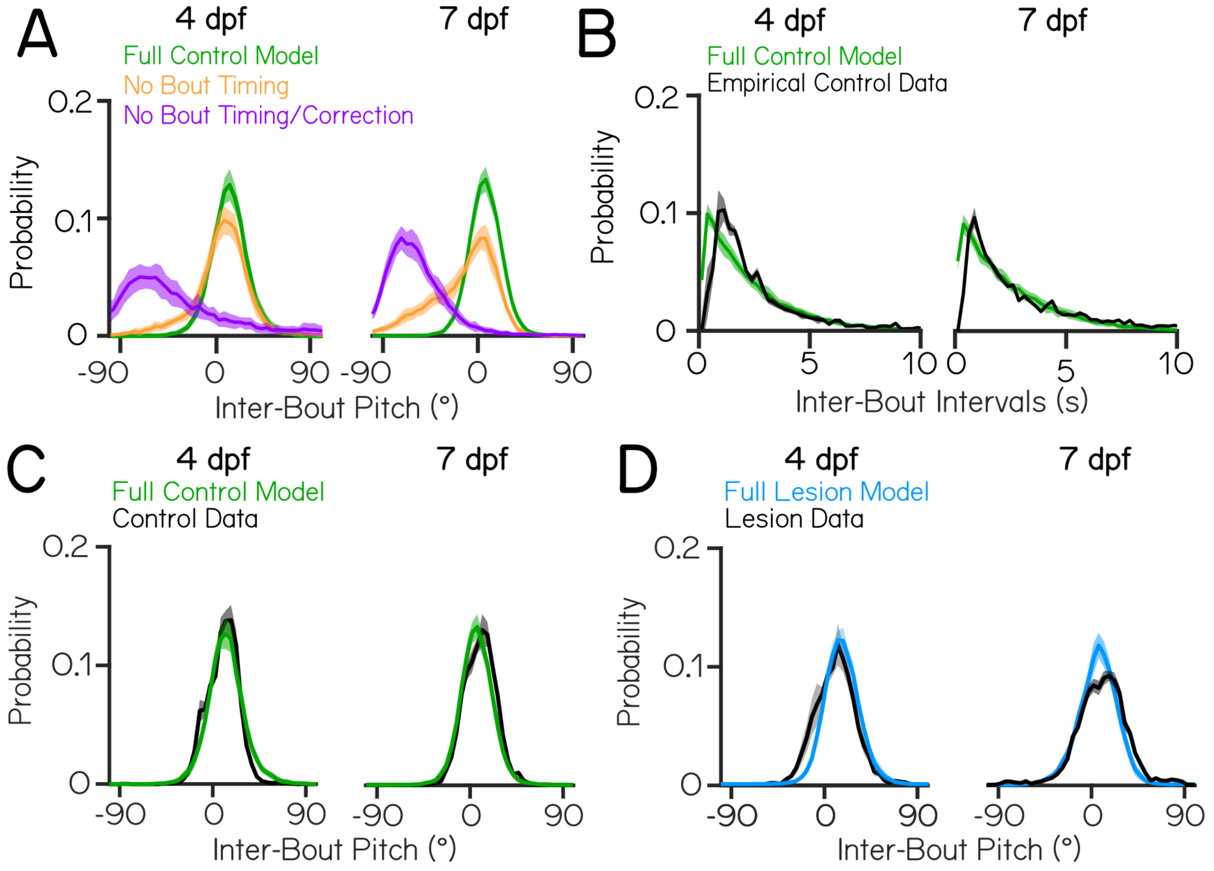

Figure S3: A model of zebrafish swimming during development generates bouts with expected pitch and event-frequency distributions. (A) Probability distributions of simulated pitch angles in full control model (green), bout timing null model (purple), and full null model (orange) are comparable for modeling data from 4 and 7 dpf, and are comparable to previous models of larval swimming[29].
(B) Probability distributions of simulated inter-bout intervals for empirical control data (black) and simulated control fish (green) using parameters derived from 4 and 7 dpf sibling control swimming datasets.
(C) Probability distributions of observed inter-bout pitch angles at 4 and 7 dpf for empirical control data (black) and simulated control fish (green) and
(D) empirical lesion data (black) and simulated lesion fish (blue)
